# Accuracy of the 6-Minute Walk Test for Assessing Functional Capacity in Patients With Heart Failure With Preserved Ejection Fraction and Other Chronic Cardiac Pathologies: Results of the ExIC-FEp Trial and a Meta-Analysis

**DOI:** 10.1186/s40798-024-00740-6

**Published:** 2024-06-18

**Authors:** Iván Cavero-Redondo, Alicia Saz-Lara, Bruno Bizzozero-Peroni, Laura Núñez-Martínez, Valentina Díaz-Goñi, Ismael Calero-Paniagua, Irene Matínez-García, Carlos Pascual-Morena

**Affiliations:** 1 CarVasCare Research Group (2023-GRIN-34459), Faculta de Enfermería de Cuenca, Universidad de Castilla-La Mancha, Cuenca, 16001 Spain; 2 Facultad de Ciencias de la Salud, Universidad Autónoma de Chile, Talca, 3460000 Chile; 3 Instituto Superior de Educación Física, Universidad de la República, Rivera, 40000 Uruguay; 4 Cardiology Service, Hospital Virgen de la Luz, Cuenca, 16001 Spain; 5 Internal Medicine Service, Hospital Virgen de la Luz, Cuenca, 16001 Spain; 6 Health and Social Research Centre, Universidad de Castilla-La Mancha, Cuenca, 16001 Spain

**Keywords:** Heart failure with preserved ejection fraction, 6-minute walk test, Cardiopulmonary exercise test, Screening accuracy

## Abstract

**Background:**

Heart diseases, particularly heart failure, significantly impact patient quality of life and mortality rates. Functional capacity assessment is vital for predicting prognosis and risk in these patients. While the cardiopulmonary exercise test is considered the gold standard, the 6-minute walk test has emerged as a more accessible alternative. However, the screening accuracy and optimal cut-off points of the 6-minute walk test for detecting severely reduced functional capacity in cardiac pathologies, including heart failure with preserved ejection fraction, are unclear. The study aimed to analyse the diagnostic accuracy of the 6-minute walk test for detecting reduced functional capacity, defined as VO_2max_ < 14 ml/kg/min, compared with the cardiopulmonary exercise test in participants with heart failure with preserved ejection fraction using data from the “Ejercicio en Insuficiencia Cardiaca con Fracción de Eyección Preservada” (ExIC-FEp) trial; and to compare these results with previous studies investigating the screening accuracy for assessing functional capacity of the 6-minute walk test in participants with other chronic cardiac pathologies through a meta-analysis.

**Results:**

The ExIC-FEp trial involved 22 participants with heart failure with preserved ejection fraction, who were not treated with beta-blockers, using the cardiopulmonary exercise test, specifically VO_2max,_ as the reference test. The 6-minute walk test had a sensitivity of 70%, a specificity of 80%, and an area under the curve of 76% in the ExIC-FEp trial. Five studies were included in the meta-analysis showing a sensitivity of 79%, a specificity of 78%, and an area under the curve of 85%.

**Conclusion:**

In conclusion, the 6-minute walk test holds promise as a screening tool for assessing functional capacity in heart failure with preserved ejection fraction and chronic heart diseases, with a VO_2max_ < 14 ml/kg/min as a reference point. It demonstrates moderate to good screening accuracy. However, the screening accuracy and optimal cut-off points of the 6-minute walk test for detecting severely reduced functional capacity, regardless of aetiology, are unclear.

**Trial Registration:**

NCT05726474. Registered 16 February 2023, https://clinicaltrials.gov/study/NCT05726474.

**Supplementary Information:**

The online version contains supplementary material available at 10.1186/s40798-024-00740-6.

## Key Points

• The 6-minute walk test (6MWT) shows promise as a screening tool for assessing functional capacity in participants with heart failure with preserved ejection fraction (HFpEF) and other chronic cardiac pathologies.

• The screening accuracy of the 6MWT was analysed in comparison to the gold standard cardiopulmonary exercise test (CPET) in participants with HFpEF from the ExIC-FEp trial. The 6MWT demonstrated a sensitivity of 70%, specificity of 80%, and an area under the curve (AUC) of 76%.

• A meta-analysis comparing the screening accuracy of the 6MWT for detecting severely reduced functional capacity across various cardiac pathologies revealed consistent results. The pooled estimates showed a sensitivity of 79%, a specificity of 78%, and an AUC of 85%.

• The 6MWT offers a cost-effective and easily implementable alternative to the CPET for assessing functional capacity. Its moderate to good screening accuracy and widespread availability make it a valuable screening tool in the screening evaluation of participants with HFpEF and other chronic cardiac pathologies.

**Supplementary Information:**

The online version contains supplementary material available at 10.1186/s40798-024-00740-6.

## Background

Cardiac pathologies represent a diverse spectrum of conditions, each with its own specific etiology, clinical manifestations, and prognostic implications. The most representative is heart failure (HF), with an incidence of 840 cases per 100,000 inhabitants per year, a prevalence of 3.4% and a one-year mortality of 24% in adults worldwide [1]. With regard to prognostic factors for the development and mortality of HF, there are several tools, such as the New York Heart Association classification and other questionnaires with a certain degree of subjectivity, which questions their usefulness for stratifying the risk of death in these patients [2]. Other more objective tests with greater predictive power include functional capacity, as determined by the cardiopulmonary exercise test (CPET), the 6-minute walk test (6MWT) and other tests [3, 4].

CPET is the gold standard for assessing functional capacity in patients with HF and other chronic cardiac pathologies [5]. CPET can be performed on a treadmill or on a bicycle, both of which have comparable exercise protocols, so the choice depends on the preferences of the participants and the health centre [6]. CPET measures maximal oxygen consumption (VO_2max_), carbon dioxide production, ventilation and the slope of carbon dioxide production per minute of ventilation [7]. Specifically, VO_2max_ is associated with survival in HF with reduced (HFrEF) and preserved ejection fraction (HFpEF) and is altered in other associated chronic cardiac pathologies, such as dilated cardiomyopathy or Chagas disease, even in the presence of normal ejection fraction [8–11]. Although there is no established cut-off point for VO_2max_ to predict survival, cut-off points of 14.0 to 15.5 ml/kg/min are usually used, exceptionally 20 ml/kg/min, depending on the specific disease and the author’s guidelines [10, 12]. It should be noted that while a cut-off of 14 ml/kg/min is commonly utilised, its validation across all cardiac pathologies, including adult congenital disease, remains an area of ongoing research and debate [13].

Although the CPET is a validated and reliable test for assessing functional capacity in these patients, it requires expensive equipment and extensive preparation by healthcare professionals and is not always well tolerated by patients. Alternative submaximal exercise tests and CPET parameters such as ratio of minute ventilation to carbon dioxide production (VE/VCO_2_) or exercise oscillatory ventilation have therefore been proposed [14]. Among the most widely used is the 6MWT, which is easier to perform, less expensive and well tolerated by patients [15]. The 6MWT is associated with VO_2max_ in this type of population and is strongly associated with other outcomes, such as mortality or cardiac events [16]. For example, 6MWT values below 300 m are associated with a decrease in survival in mild (mild fatigue or shortness of breath with strenuous physical activity) to moderate (increased limitation of physical activity due to fatigue and dyspnoea) HF (17–18), as well as a 55% increase in cardiovascular events per 104-metre decrease in stable coronary artery disease [19].

As mentioned above, functional capacity as measured by the CPET and 6MWT is associated with chronic cardiac pathologies. Both are also correlated with mortality. However, the 6MWT cut-off points for better or worse prognosis are not well established [20]. Thus, the aims of this study were (i) to analyse the screening accuracy for assessing functional capacity of the 6MWT compared with CPET in participants with HFpEF from the “*Ejercicio en Insuficiencia Cardiaca con Fracción de Eyección Preservada”* (ExIC-FEp) trial, focusing on sensitivity, specificity, area under curve (AUC), positive and negative likelihood ratio (PLR and NLR); and (ii) to compare these results with previous studies investigating the screening accuracy for assessing functional capacity of 6MWT compared to CPET in participants with other chronic cardiac pathologies through a meta-analysis.

## Methods

### ExIC-FEp Trial Data Analysis

#### Study Design

This study is a cross-sectional study whose sample was obtained from the baseline data of participants in the ExIC-FEp trial, which followed the STARD 2015 guidelines [21]. The ExIC-FEp trial is a single-blind, randomised clinical trial conducted in the province of Cuenca (Spain) to compare the efficacy of combined exercise (aerobic and strength training) and high-intensity interval training on functional capacity, diastolic function, endothelial function and arterial stiffness in participants with HFpEF. The ExIC-FEp trial followed the Declaration of Helsinki, was previously approved by the Clinical Research Ethics Committee of the Cuenca Health Area (REG: 2022/PI2122) and registered at ClinicalTrials.gov (NCT05726474). The full protocol has previously been published elsewhere [22].

#### Study Sample

Of the 76 subjects enrolled in the ExIC-FEp trial, we included the baseline values of 22 participants for this analysis, selecting only subjects not treated with beta-blockers and enrolled between January 2023 and March 2024. Participants were informed of the purpose and objectives of the study, and informed consent was obtained. A cardiologist determined whether the participants were able to exercise.

#### Reference Standard

CPET was used as the gold standard. CPET was performed with an Ergoline600 bicycle ergometer and a gas analyser (K5 COSMED). This test was used to determine VO_2max_, with a cut-off of 14 mL/kg/min, as well as the ventilation slope between carbon dioxide (VE/CO_2_ slope), effort time and achieved workload. CPET was incremental or progressive, and participants were monitored with a 12-lead electrocardiogram [23].

#### Index Test

The 6MWT was used as the index test. Two signs were placed in a 30-metre corridor (flat surface). This corridor consists of a distance of 29 m between two markers placed at each end of the corridor, with an additional 0.5 m at each end to allow the participant to turn comfortably. Therefore, the total length of the corridor, including the additional space for turning, is 30 m [24]. Participants were instructed to walk back and forth along the corridor for six minutes, with the turning point marked at each end. It is important to note that turning at each end of the corridor may affect the participant’s performance during the test. The act of turning requires participants to decelerate as they approach the marker, perform a turn, and then accelerate again to continue walking. This change in walking rhythm and gait pattern could potentially disrupt participants’ pace and overall performance [25]. Participants were accompanied by one of the two examiners (immediately behind them) and were encouraged to keep up the pace every minute by standardised feedback. The patient’s dyspnoea was assessed using the Borg scale. A second examiner monitored heart rate using a Polar H10 heart rate monitor. The Borg scale and heart rate were evaluated at baseline, every minute and at the end of the test. The Borg scale is a subjective rating scale used to assess the level of dyspnoea, or shortness of breath, experienced by the patient during physical activity. It consists of a numerical rating from 0 to 10, where 0 represents no dyspnoea at all and 10 represents the most severe dyspnoea imaginable [26]. The main result of the test is the final distance walked.

#### Other Variables

Age and sex were obtained by direct questioning. N-terminal pro-brain natriuretic peptide (NT-proBNP) was determined by enzyme-linked immunosorbent assay (R & D Systems, Minneapolis, MN, US). VO_2max_, VE/CO_2_ slope, exercise time and achieved workload were determined by the CPET. Left ventricular ejection fraction (LVEF), early/atrial filling velocity ratio (EA) and early mitral/mitral annular velocity ratio (E/e′) were determined by echocardiography and Sonosite SII Doppler ultrasound (Sonosite Inc., Bothell, WA, US).

### Meta-Analysis

A systematic review with meta-analysis was conducted according to the Cochrane Collaboration Handbook and the Preferred Reporting Items for Systematic Reviews and Meta-Analyses (PRISMA) guidelines [27, 28]. The protocol was previously registered in PROSPERO (CRD42023441551).

#### Search Strategy

A systematic search was performed in Medline (via PubMed), Scopus, Web of Science, and the Cochrane Library, and an open search in grey literature, including Google Scholar, Theseo, Networked Digital Library of Theses and Dissertations, and Open Grey, from inception to July 2023. The systematic search included the terms heart failure, heart disease, coronary artery disease, cardiovascular disease*, VO2peak, peak oxygen, peak VO2, peak oxygen uptake, peak oxygen consumption, six-minute walking test, walk* test*, six-minute, 6MWD, and 6MWT. References of included studies and previous reviews were checked. The search strategy is described in detail in Supplementary material Appendix S1.

The literature search was conducted independently by two reviewers (IC-R and CP-M), and disagreements were resolved by consensus or by a third reviewer (AS-L).

#### Inclusion/Exclusion Criteria

Inclusion criteria were as follows: (1) participants: population with chronic cardiac pathologies, including heart failure, dilated cardiomyopathy and Chagas disease; (2) design: cross-sectional studies, including cross-sectional studies of longitudinal studies; (3) screening method: The reference standard was CPET determining VO_2max_ using a treadmill or bicycle with a cut-off of 14 to 20 mL/kg/min, while the index test was the distance walked in 6 min using the 6MWT; (4) outcome: sensitivity, specificity, AUC, PLR, and NLR obtained using receiver operating characteristic (ROC) curves. There were no language restrictions.

Exclusion criteria were as follows: (1) participants: studies that included participants with chronic and acute cardiac pathologies in the analysis and that could not extract results separately for participants with chronic cardiac pathologies; (2) design: studies with underage participants.

Study selection was conducted independently by two reviewers (IC-R and CP-M), and disagreements were resolved by consensus or by a third reviewer (AS-L).

#### Data Extraction

An ad hoc table was created with the data extracted from the included studies and included (1) reference (author and year of publication); (2) country in which the study was conducted; (3) sample size (total, male, female); (4) mean age of participants; (5) type of chronic cardiac pathologies; (6) description of the reference standard and index test; and (7) outcome: 6MWT cut-off, sensitivity, specificity, AUC, PLR, NLR.

#### Risk of Bias Assessment

Risk of bias was assessed using the Quality Assessment of Diagnostic Accuracy Studies (QUADAS 2) tool [29]. This tool assesses four domains, including patient selection, index test, gold standard, and flow and timing. This tool also assesses the applicability of the tool. Each item is rated as high risk, unclear risk or low risk.

The risk of bias assessment was conducted independently by two reviewers (ICR and CP-M), and disagreements were resolved by consensus or by a third reviewer (AS-L).

### Statistical Analysis

#### ExIC-FEp Data Analysis

The normality of continuous variables was tested using normal probability plots and the Kolmogorov‒Smirnov test. Continuous descriptive data were expressed as the means and standard deviations. The existence of statistically significant differences in descriptive variables between the sexes was tested using Student’s t test.

A linear regression was performed between VO_2max_ and 6MWT to obtain the slope of the regression and the coefficient of determination (R^2^). An ROC curve was then generated using VO_2max_ with a cut-off of 14 mL/kg/min as the reference standard test and the 6MWT as the index test. Sensitivity and specificity were determined using the Youden index, and the AUC, PLR and NLR were also estimated.

Statistical analyses were performed using IBM SPSS 28 (SPSS Inc., Chicago, IL).

#### Meta-Analysis

A narrative synthesis of the results of each study was performed. Subsequently, random effects meta-analyses of sensitivity, specificity, AUC, PLR and NLR obtained from ROC curves were performed, including data from the ExIC-FEp trial [30]. Heterogeneity was assessed using the *I*^2^ statistic and classified as not important if < 30%, moderate if 30–50%, substantial if 50–75%, considerable if > 75%, and considered statistically significant if *p* < 0.05 [27]. Publication biases of sensitivity and specificity were assessed visually and using Egger’s test and were statistically significant if *p* < 0.10 [31].

Statistical analyses were performed using Stata v15 (StataCorp, College Station, TX, US).

## Results

### Results of the ExIC-FEp Trial

#### Characteristics of the Participants

The study included 22 participants, of whom 10 were male and 12 were female. The mean age of the participants was 72.5 ± 7.0 years. There were no statistically significant differences between sexes in most of the descriptive variables, such as VO_2max_, VE/CO_2_ slope, achieved workload, NT-proBNP, 6MWT, EA and E/e’. However, males had higher age and values for exercise time and females had higher LVEF. The full description of the participants’ characteristics can be found in Table 1.

**Table 1:** Baseline characteristics of the participants included in the ExIC-FEp study

Variable	Total	Male	Female	*p*-value
Sample size	22	10	12	-
Age (years)	72.5 ± 7.0	75.7 ± 7.3	69.8 ± 5.8	0.048*
VO_2max_	15.0 ± 4.6	14.6 ± 3.0	15.4 ± 5.6	0.672
VE/CO2 slope	32.4 ± 7.1	31.5 ± 6.7	33.2 ± 7.8	0.605
Exercise time	5.7 ± 2.6	7.1 ± 2.9	4.6 ± 1.6	0.020*
Achieved workload	44.9 ± 20.6	51.9 ± 23.5	39.1 ± 16.6	0.150
NT-proBNP	528.5 ± 698.6	760.6 ± 664.1	335.0 ± 693.6	0.160
6MWT	363.5 ± 114.3	317.9 ± 110.1	401.5 ± 107.5	0.088
LVEF	58.5 ± 6.9	52.8 ± 4.6	62.6 ± 5.1	< 0.001*
EA	1.1 ± 0.7	1.3 ± 1.1	0.9 ± 0.2	0.323
E/e′	9.5 ± 3.4	8.8 ± 2.3	10.1 ± 4.2	0.402

Abbreviations: VO_2max_ (maximal oxygen consumption); NT-proBNP (Pro-brain natriuretic peptide); 6MWT (six-minutes walking test); LVEF (left ventricular ejection fraction); EA (early/atrial filling velocity ratio); e’ (early mitral annular velocity); E/e’ (early mitral/mitral annular velocity ratio). * *p* < 0.05

#### Comparability Between CPET and 6MWT and ROC Curve

**Fig. 1:**
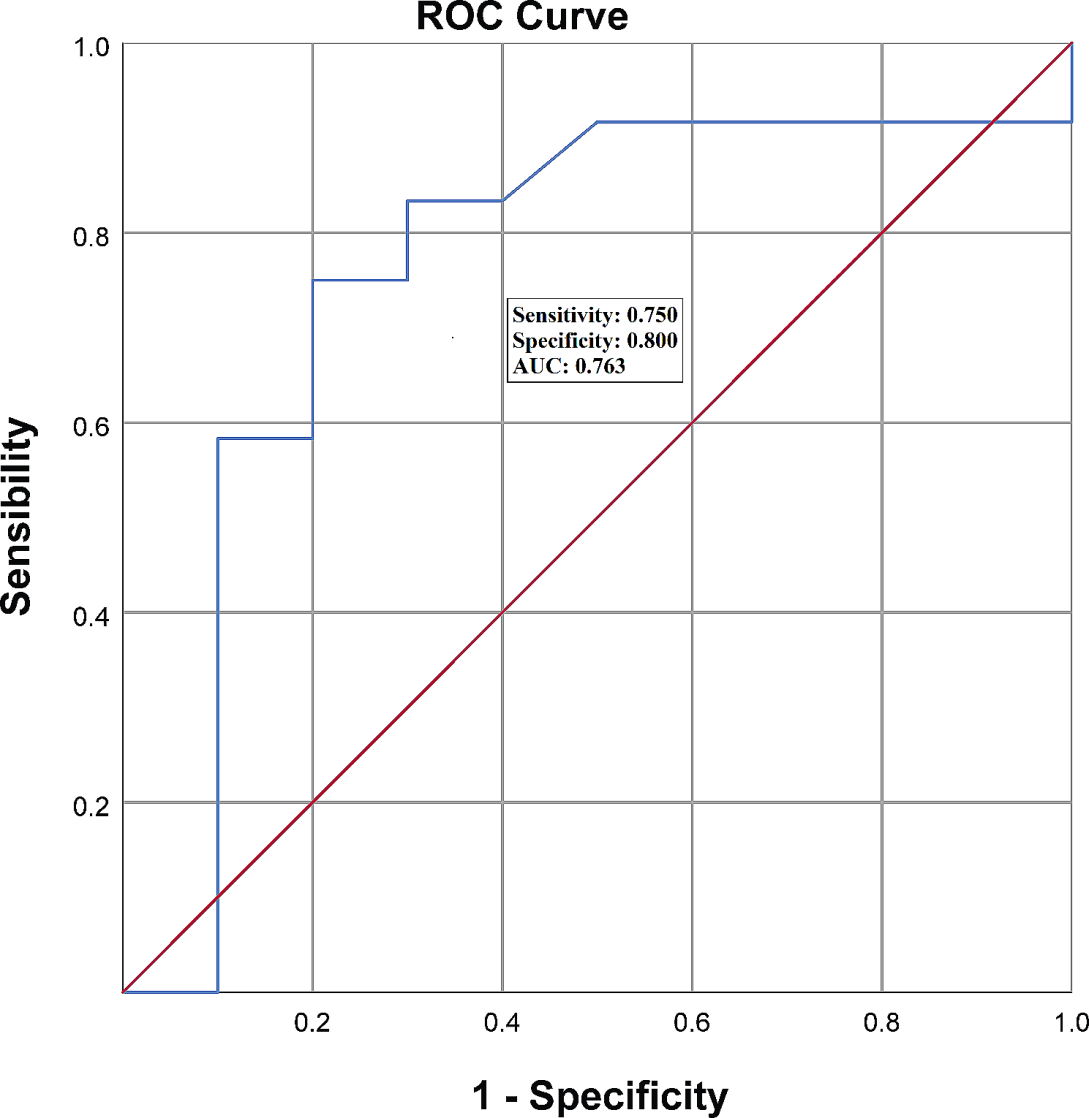
Receiver operating characteristic (ROC) curve for the comparison of the 6-minute walk test (6MWT) and the cardiopulmonary exercise test (CPET) to detect severely reduced functional capacity (VO_2max_ < 14 mL/kg/min)

Supplementary Fig. S1 shows the linear regression between CPET and 6MWT. The slope of the line was 8.25, and the constant was 239, with an R^2^ = 0.108 (*R* = 0.329). Fig. 1 shows the ROC curve using the CPET as the reference standard and the 6MWT as the index test. According to the results, the maximum Youden index was 0.550, giving a sensitivity of 0.75 and a specificity of 0.80, which was the cut-off for the 6 WMT of 358,50 m. The area under the ROC curve was 0.76 [95% confidence interval (95% CI): 0.54, 0.99 the PLR was 3.75 and the NLR was 0.31.

### Meta-Analysis

Of the 911 studies identified, four met the inclusion criteria [32–35] and were included in the systematic review and meta-analysis along with the ExIC-FEp trial, while six studies were excluded with justification (Supplementary Fig. S2, Table 2, Supplementary Table S1).

**Table 2:** Baseline characteristics of participants in the studies included in the systematic review

Reference	Country	Sample			Age	HF type	Outcome					
		Total	Male	Female			Cut-off (m)	Sens	Spec	AUC	PLR	NLR
Cavero-Redondo et al. [22]	Spain	22	10	12	72.5 ± 7.0	HF with EF preserved	359	0.75	0.80	0.76	3.75	0.31
Costa et al. [32]	Brazil	35	23	12	47.1 ± 8.2	Chagas disease	520	0.75	0.71	0.77	2.59	0.35
Kehmeier et al. [33]	Germany	102	51	51	35.4 ± 13.6	Congenital heart disease	482	0.79	0.76	0.87	3.29	0.28
Morales et al. [34]	Spain	100	80	20	53.0 ± 10.0	HF with EF < 40%	450	0.80	0.83	0.83	4.71	0.24
Pulz et al. [35]	Brazil	63	44	19	51.3 ± 10.2	HF with EF < 35% (some with Chagas disease)	490	0.83	0.83	0.89	4.88	0.20

Abbreviations: HF: heart failure; m: meters; Sens; sensitivity; Spec: specificity; AUC: area under curve; PLR: positive likelihood ratio; NLR: negative likelihood ratio; HFpEF: heart failure with preserved ejection fraction; EF: ejection fraction

Of the five included studies (four included in the search and the ExIC-FEp trial data), two were conducted in Brazil, two in Spain and one in Germany. A total of 322 participants (208 male and 114 female) were included, with a mean age ranging from 35.4 to 72.5 years. Two studies were conducted in participants with HFrEF, one study in participants with HFpEF, one study in participants with congenital heart disease, and one study in participants with Chagas disease. Different protocols were used for the CPET and 6MWT, which are described in Supplementary Table S2.

#### Risk of Bias Assessment

According to the QUADAS-2 tool, 2 out of 5 (40%) studies had a high risk of bias in the domain of flow and timing, 3 out of 5 (60%) studies had an unclear risk in the domain of patient selection, and 1 out of 5 (20%) studies had an unclear risk in the domain of index test and reference standard. For applicability, 3 out of 5 (60%) studies had unclear risk in the domain of patient selection. The risk of bias assessment is described in Supplementary Fig. S3.

#### Systematic Review and Meta-Analysis

Supplementary Table S3 summarises the results of the cut-off of the 6MWT, sensitivity, specificity, AUC, PLR and NLR using the 6MWT as index tests and the CPET as the reference standard. The cut-off ranged between 359 and 520 m, sensitivity between 0.75 (95% CI: 0.39, 1.00) and 0.83 (95% CI: 0.69, 0.97), specificity between 0.71 (95% CI: 0.53, 0.89) and 0.83 (95% CI: 0.67, 0.99), AUC between 0.76 (95% CI: 0.54, 0.99) and 0.89 (95% CI: 0.75, 1.03), PLR between 2.59 (95% CI: 0.21, 31.42) and 4.88 (95% CI: 0.49, 48.61), and NLR between 0.20 (95% CI: 0.02, 2.04) and 0.37 (95% CI: 0.03, 4.07).

**Fig. 2:**
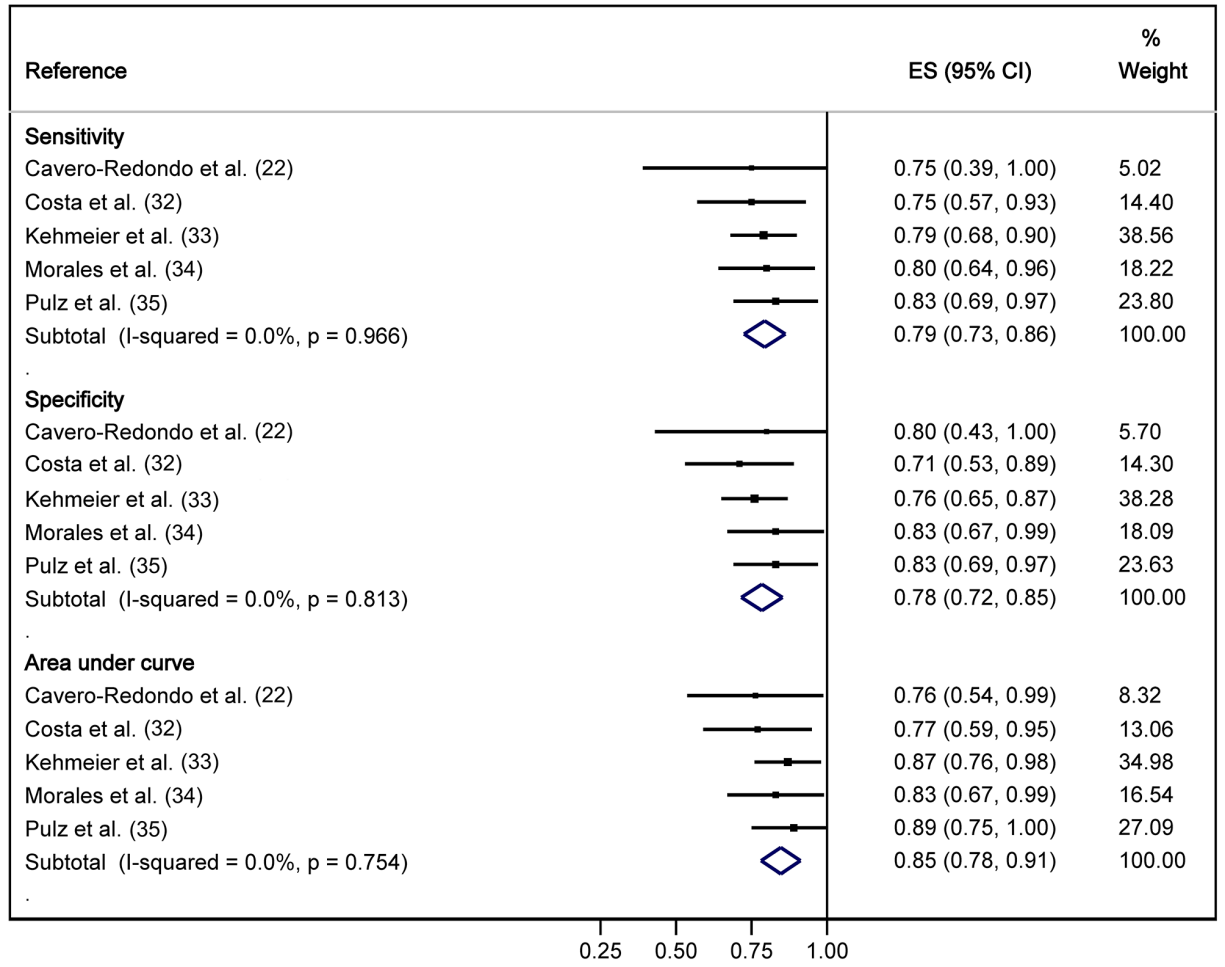
Forest plot for the sensitivity, specificity and area under the curve (AUC) of the 6-minute walk test (6MWT) to detect severely reduced functional capacity (VO_2max_ < 14 mL/kg/min)

**Fig. 3:**
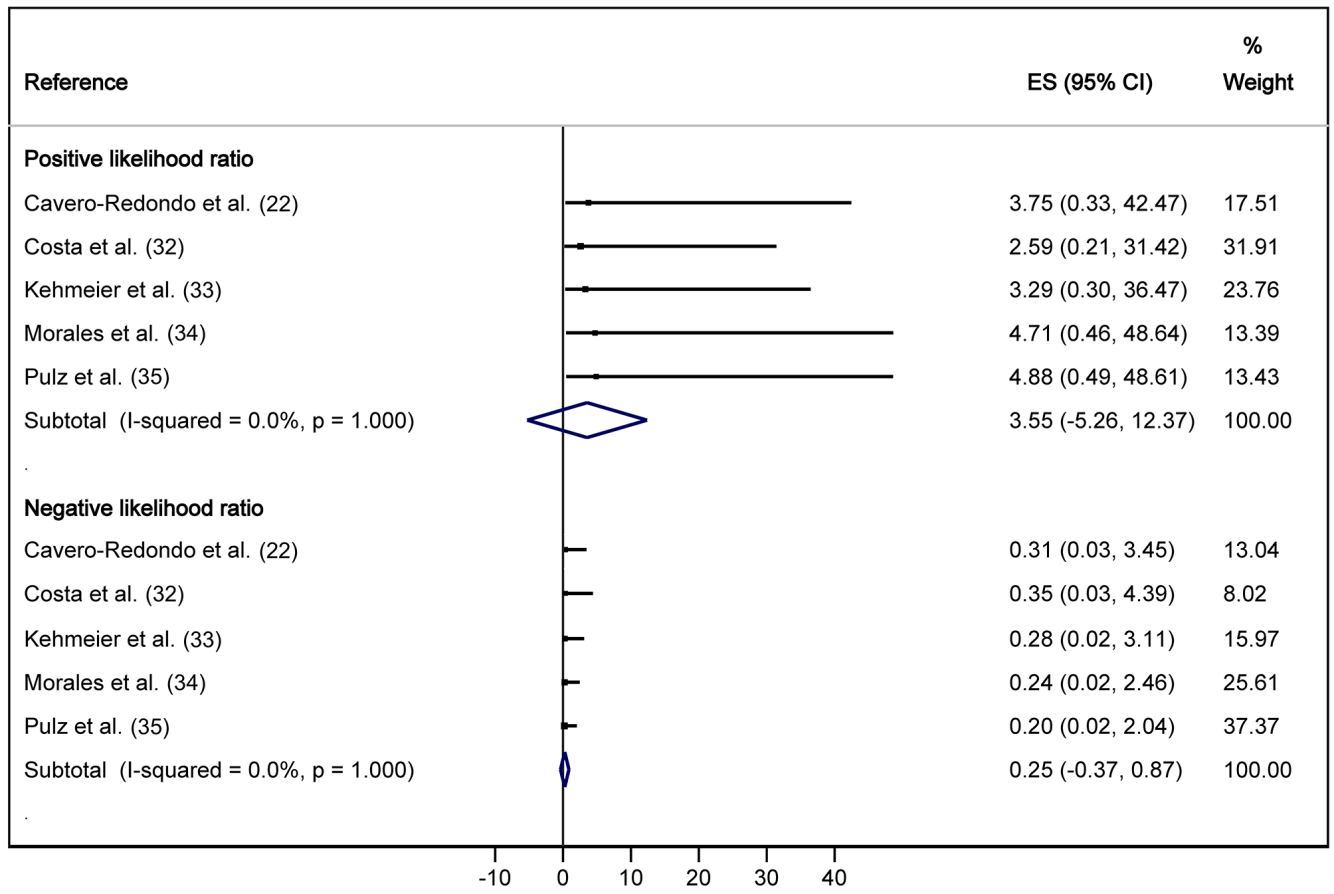
Forest plot for the positive and negative likelihood ratio (PLR and NLR) of the 6-minute walk test (6MWT) to detect severely reduced functional capacity (VO_2max_ < 14 mL/kg/min)

Pooled estimates from meta-analyses showed a sensitivity of 0.79 (95% CI: 0.73, 0.86), specificity of 0.78 (95% CI: 0.72, 0.85), AUC of 0.85 (95% CI: 0.72, 0.85), PLR of 3.55 (95% CI: -5.26, 12.37) and NLR of 0.25 (95% CI: -0.37, 0.87). In all cases, heterogeneity was not important and not statistically significant (*I*^2^ = 0.0, *p* > 0.05) (Figs. 2 and 3). There was publication bias either visually (Supplementary Figs. S4-S8) or by Egger’s test for AUC (*p* = 0.074) for PLR (*p* = 0.012) and for NLR (*p* = 0.032).

## Discussion

The present study analysed the screening accuracy for assessing the functional capacity of the 6MWT compared to the CPET in participants with HFpEF from the ExIC-FEp trial, focusing on sensitivity, specificity, AUC, PLR, and NLR. Additionally, these findings were compared with previous studies investigating the screening accuracy for assessing the functional capacity of the 6MWD compared to the CPET in participants with other chronic cardiac pathologies through a meta-analysis. The results obtained from the ExIC-FEp trial demonstrated a sensitivity of 69%, specificity of 84%, and an AUC of 75% with a cut-off point of 359 m in the 6MWT. When considering the meta-analysis results, a sensitivity of 78%, specificity of 79%, and an AUC of 84% were observed. These findings provide valuable insights into the screening utility of the 6MWT for assessing functional capacity in the context of HFpEF and highlight its potential comparability in different chronic cardiac pathologies.

The diversity of cardiac pathologies present in our sample underscores the complexity of assessing functional capacity in these patients. The variability in underlying aetiologies, clinical manifestations and response to treatment among conditions such as HFpEF, hypertrophic cardiomyopathy, dilated cardiomyopathy, and cardiac amyloidosis poses significant challenges to the interpretation of results. Our study highlights the inherent complexity of assessing functional capacity across a spectrum of cardiac pathologies. The use of VO_2max_ thresholds, particularly the commonly used threshold of 14 ml/kg/min, requires nuanced interpretation given its variability across different cardiac pathologies. Although historically studies such as the seminal work of Mancini et al. in 1991 [10] laid the groundwork for the establishment of these thresholds, subsequent research has highlighted the need for a more tailored approach that considers the heterogeneity of cardiac diseases [36]. Furthermore, the evolving landscape of pharmacological interventions, including the widespread use of beta-blockers, underscores the need to review and refine these thresholds [37]. In addition, our findings highlight the central role of CPET in elucidating exercise limitations, particularly in conditions such as HF-PEF, where its superiority over alternative modalities such as 6MWT is well documented [38].

Furthermore, the inherent complexity of assessing functional capacity in participants with chronic cardiac pathologies must be considered, particularly in the context of HFpEF, where phenotypic heterogeneity is remarkable [38]. While this study focused on functional capacity assessment for the screening utility of the 6MWT in participants with HFpEF, this group encompasses a wide range of aetiologies and clinical presentations. For example, within the cohort of patients with HFpEF there may be patients with cardiac amyloidosis who have a preserved ejection fraction and who may experience a significant reduction in functional capacity [39, 40]. This phenomenon highlights the limitations of relying solely on resting LVEF as a predictor of exercise capacity and emphasises the need for a more comprehensive assessment of cardiac function and functional capacity in these patients [41, 42]. Thus, it is important to consider the diversity of chronic cardiac pathologies when interpreting our findings and designing future assessment and treatment strategies.

The variability of CPET values and the differences between bicycle and treadmill testing modalities are key considerations when interpreting the diagnostic accuracy of functional capacity assessments in patients with HFpEF and other chronic cardiac pathologies, as investigated in our study. The wide range of VO_2max_ values, particularly in the 14–20 ml/kg/min range, highlights the complexity of these assessments and the challenges they pose in certain clinical contexts [43]. Additionally, the discrepancy in values between treadmill and cycle testing, attributed to differences in muscle recruitment, warrants careful consideration. As highlighted, treadmill testing often engages more muscle groups, potentially resulting in higher VO_2max_ values compared to cycle testing [44].

The applicability and clinical significance of sensitivities and specificities, considering the results of the ExIC-FEp trial and the meta-analysis, as well as the PLR and NLR, should be carefully evaluated in the context of the 6MWT. The ExIC-FEp trial demonstrated a sensitivity of 75%, indicating that the test correctly identified 8 out of 10 participants with severely reduced functional capacity (VO_2max_ < 14 mL/kg/min). The specificity of 80% implies that 8 out of 10 individuals with non-severely reduced functional capacity were correctly classified as negative. These values indicate the ability of the test to accurately detect true positive cases and exclude individuals with non-severely reduced functional capacity (VO_2max_ ≥ 14 mL/kg/min). Moreover, the ExIC-FEp trial revealed a PLR of 3.75, suggesting that individuals with severely reduced functional capacity (VO_2max_ < 14 mL/kg/min) are 3.75 times more likely to have a positive test result than those with non-severely reduced functional capacity (VO_2max_ ≥ 14 mL/kg/min). An NLR of 0.31 indicates that individuals with non-severely reduced functional capacity (VO_2max_ < 14 mL/kg/min) are 0.31 times as likely to have a negative test result compared to those with severely reduced functional capacity (VO_2max_ ≥ 14 mL/kg/min). Similarly, the meta-analysis showed a sensitivity of 79%, specificity of 78%, PLR of 3.55, and NLR of 0.25. These values provide additional insights into the screening performance of the 6MWT in various chronic cardiac pathologies. However, it is important to interpret these sensitivity, specificity, PLR, and NLR values together with other performance measures, such as positive and negative predictive values, to fully assess the screening value of the test [45]. This comprehensive assessment facilitates informed decision-making, enables risk stratification, and optimises patient care [46].

Based on these values and considering established standards [17], it can be determined that the 6MWT has clinical implications as a screening tool. The moderate to good AUC values and acceptable sensitivity and specificity suggest that the test may serve as a valuable screening tool to identify individuals with an increased likelihood of severely reduced functional capacity (VO_2max_ < 14 mL/kg/min) amongst patients with chronic cardiac pathologies. However, it is important to acknowledge that the classification of the test may depend on specific clinical settings, disease prevalence in the target population, and other relevant factors [47]. The clinical implications of the test should also consider issues such as the availability of effective treatments, associated costs, and acceptability to patients and healthcare professionals [48, 49].

The wide range of 6MWT cut-off values identified in our meta-analysis, ranging from 359 to 520 m, underscores the need for further evaluation of the clinical utility of this test in patients with HF-PEF and other chronic cardiac pathologies. This variability may be due to several factors. First, the heterogeneity of the participants included in the studies, including differences in age, sex, comorbidities, disease severity and medication regimens, significantly influences the test results [50]. In addition, differences in inclusion and exclusion criteria between studies, particularly with regard to the severity and stability of the underlying heart disease, contribute to the observed variability in cut-off values. Furthermore, differences in test administration protocols, such as walking instructions, pacing techniques, rest periods and environmental conditions, introduce additional sources of variability [51]. Inconsistencies in these aspects of the procedure may affect patient motivation, the effort expended during the test and, ultimately, the distance covered [52]. For example, differences in the stimulus provided during the test, such as verbal cues or motivational feedback, may influence the patient’s performance and, consequently, the distance measured [53]. In addition, variations in the test environment may influence patient engagement and test results [54]. Therefore, a critical discussion on the standardisation of patient selection criteria, test administration protocols and environmental conditions is essential to minimise variability and improve the reliability and validity of the 6MWT as a tool to assess functional capacity in patients with HF-PEF and other chronic cardiac pathologies. Future research should focus on identifying and addressing these sources of variability using standardised protocols and rigorous methodological approaches to ensure consistency and reproducibility of test results across studies. Furthermore, given the considerable variation in cut-off values and diagnostic performance measures between studies, additional research is warranted to provide more conclusive evidence regarding the reliability of the 6MWT as an independent measure of functional capacity in this patient population.

In addition, although our study included baseline values for 22 of the 76 participants in the ExIC-FEp trial who were not treated with beta-blockers, this specific selection was intended to mitigate potential confounding effects of beta-blocker treatment on functional capacity assessment using the 6MWT. Beta-blocker therapy is a cornerstone of heart failure treatment, but its effect on exercise capacity in HFpEF remains an area of interest [55]. HFpEF is characterised by increased left atrial and left ventricular end-diastolic pressures and reduced ventricular compliance. Thus, exercise-induced increase of cardiac output (CO) is achieved at higher filling pressures and oxygen demand, which is the substrate of exertional dyspnoea [56]. Evidence suggests that beta-blockers may reduce the chronotropic response and exercise capacity in HFpEF, which could affect outcomes assessed by CPET, such as VO2max and increased oxygen pulse [42]. By focusing on patients not receiving beta-blocker therapy, we aimed to isolate the effects of HFpEF itself on functional capacity and provide a clearer understanding of the role of the 6MWT in this population. Further research into the effect of beta-blockers on 6MWT performance in HFpEF supports our decision to exclude patients on beta-blocker therapy from our analysis, thereby improving the validity and interpretability of our results.

When comparing our results with those of previous studies, several important considerations emerge. For example, one study highlighted the importance of stride length on 6MWT performance in HF patients, emphasising the need to consider biomechanical and technical variables during testing that may influence the distance walked and consequently its usefulness as a tool to assess functional capacity [57]. In addition, other research highlighted the relationship between the 6MWT and health-related quality of life in HF patients, suggesting that the 6MWT serves not only as a measure of functional capacity, but also as an indicator of patients’ perceptions of their health status and well-being [58]. Conversely, another study identified predictors of 6MWT performance in HF patients, improving our understanding of the determinants of functional capacity in this population [59]. Finally, a randomised trial highlighted the importance of exercise training in improving functional capacity, as measured by the 6MWT, in elderly people with HFpEF [60]. Taken together, these findings complement our results and provide a broader perspective on the role of the 6MWT in the assessment and management of patients with HF, highlighting its clinical utility as an assessment tool and a means of monitoring treatment and rehabilitation progress.

### Strengths and Limitations

Our study presents several strengths that contribute to its significance and reliability. First, the study addresses a critical need in the field of cardiology by investigating the diagnostic accuracy of the 6MWT for assessing functional capacity, particularly in patients with HFpEF and other chronic cardiac pathologies. This will fill a gap in the knowledge of alternative assessment tools for functional capacity beyond the gold standard CPET. Second, the inclusion of the ExIC-FEp trial, a well-designed and rigorously conducted clinical trial, provides robust primary data on the diagnostic accuracy of the 6MWT in participants with HFpEF. The study adhered to established guidelines, ensuring methodological rigour and reliability of the results. In addition, the meta-analysis conducted to synthesise data from multiple studies adds depth and breadth to the research, allowing for a comprehensive assessment of the diagnostic accuracy of the 6MWT in different chronic cardiac pathologies. The systematic review process followed rigorous methodologies, including a thorough search strategy and quality assessment of the included studies, which enhances the credibility of the meta-analysis results.

Additionally, several limitations of this study should be acknowledged. First, this study adopted a cross-sectional design, which only captured baseline data from a subset of participants enrolled in the ExIC-FEp trial. Consequently, establishing causal relationships or examining changes over time is restricted [61]. Future studies incorporating longitudinal follow-up would provide a more comprehensive understanding of the screening accuracy of the 6MWT. Second, the reliance on this subset of participants potentially limits the generalisability of our findings. The sample size could also affect the precision of the estimates [61], warranting caution when extrapolating the results to the broader population of patients with HFpEF. Third, because the ExIC-FEp trial used data from a randomised clinical trial, there is a possibility of selection bias in participant inclusion. The eligibility criteria and recruitment process used in the clinical trial may have introduced bias, thus affecting the representativeness of the study sample [62]. Fourth, the screening accuracy for functional capacity of the 6MWT may vary in different patient populations and settings, which should be considered when interpreting the findings. Fifth, a relatively small number of studies were included in our systematic review and meta-analysis. With only four studies meeting our inclusion criteria, the breadth and depth of evidence available for drawing conclusions about the benefits of the 6MWT compared to CPET may be constrained. Sixth, our study only included subjects who were not treated with beta-blockers, and the data regarding beta-blocker usage in the other studies included were not available. This lack of uniformity in subject characteristics across the studies may have introduced bias and limited the generalizability of our findings. Finally, the meta-analysis conducted to compare the screening accuracy of the 6MWT in different chronic cardiac pathologies is susceptible to potential publication bias. The inclusion of published studies may introduce a bias towards studies with significant results, potentially influencing the overall estimate of screening accuracy [63]. These limitations should be taken into account when interpreting the results, and future research should aim to address these challenges for a more robust assessment of the screening utility of the 6MWT.

## Conclusions

Based on the results obtained in the ExIC-FEp trial and the meta-analysis, it can be concluded that the 6MWT holds promise as a screening tool for assessing functional capacity in patients with HFpEF and other chronic cardiac pathologies when there is no access to CPET measurement equipment. These findings suggest that the test can be a useful tool in the screening of severely reduced functional capacity in this patient population. However, it is important to be aware of the limitations of the test, such as its moderate sensitivity and the potential for false-negative and false-positive results. In some cases, complementary screening tests for assessing functional capacity, such as CPET, may be necessary to complete the screening evaluation of severely reduced functional capacity. Furthermore, comparison with previous studies through the meta-analysis revealed consistent screening accuracy in various chronic cardiac pathologies, indicating the potential generalisability of the 6MWT in different patient populations. Overall, the 6MWT holds promise as a cost-effective and easy-to-apply tool in the screening of severely reduced functional capacity of HFpEF, but further research is warranted to validate its clinical utility and to establish optimal cut-off values for accurate diagnosis.

### Electronic Supplementary Material

Below is the link to the electronic supplementary material.

Supplementary Material 1

## Data Availability

Data and materials related to the manuscript are available on request from the corresponding author.

## Abbreviations

6MWT: 6-minute walk test
AUC: area under curve
CPET: cardiopulmonary exercise test
EA: early/atrial filling velocity ratio
E/e′: early mitral/mitral annular velocity ratio
ExIC-FEp trial: “Ejercicio en Insuficiencia Cardiaca con Fracción de Eyección Preservada” trial (“Exercise in Heart Failure with Preserved Ejection Fraction” trial)
HF: heart failure
HFpEF: heart failure with preserved ejection fraction
HFrEF: heart failure with reduced ejection fraction
LVEF: left ventricular ejection fraction
NLR: negative likelihood ratio
NT-proBNP: N-terminal pro-brain natriuretic peptide
PLR: positive likelihood ratio
PRISMA: Preferred Reporting Items for Systematic Reviews and Meta-Analyses
QUADAS 2: Quality Assessment of Diagnostic Accuracy Studies tool
ROC: receiver operating characteristic curves
VE/VCO_2_: ratio of minute ventilation to carbon dioxide production
VE/CO_2_ slope: ventilation slope between carbon dioxide
VO_2max_: maximal oxygen consumption
