# Supplementary material for: Accuracy of the 6-Minute Walk Test for Assessing Functional Capacity in Patients With Heart Failure With Preserved Ejection Fraction and Other Chronic Cardiac Pathologies: Results of the ExIC-FEp Trial and a Meta-Analysis
Source: Sports Med Open. 2024 Jun 18;10:74. doi: 10.1186/s40798-024-00740-6 (PMC11183033; doi:10.1186/s40798-024-00740-6)
Supplement: Supplementary file 1 — Supplementary Material 1 [file 40798_2024_740_MOESM1_ESM.docx]

**INDEX**

**Table S1.** Excluded studies with justified reasons.

**Table S2.** CPET and 6MWT protocols in each study.

**Table S3.** Results of the studies included in the systematic review.

**Figure S1.** Linear regression between CPET and 6MWT scores.

**Figure S2.** PRISMA flowchart of study selection.

**Figure S3.** Risk of bias assessment.

**Figure S4.** Publication bias assessment of sensitivity.

**Figure S5.** Publication bias assessment of specificity.

**Figure S6.** Publication bias assessment of area under curve (AUC).

**Figure S7.** Publication bias assessment of positive likelihood ratio (PLR).

**Figure S8.** Publication bias assessment of negative likelihood ratio (NLR).

**Appendix S1.** Search strategy.

**Table S1.** Excluded studies with justified reasons.

| **Reference** | **Main reason** |
| --- | --- |
| Corra et al. (1) | Not outcome of interest |
| Maldonado-Martín et al. (2) | Not outcome of interest |
| Meyer et al. (3) | Not design of interest |
| Qu et al. (4) | Not population of interest |
| Ritt et al. (5) | Not index test of interest |
| Węgrzynowska-Teodorczyk et al. (6) | Not outcome of interest |

Supplementary references

1. Corrà U, Giordano A, Marcassa C, Gambarin FI, Gnemmi M, Pistono M. Prognostic value of 6-min walk test compared to cardiopulmonary exercise test in patients with severe heart failure. J Cardiovasc Med (Hagerstown). 2022 Jun 1;23(6):379-386.
2. Maldonado-Martín S, Brubaker PH, Eggebeen J, Stewart KP, Kitzman DW. Association Between 6-Minute Walk Test Distance and Objective Variables of Functional Capacity After Exercise Training in Elderly Heart Failure Patients With Preserved Ejection Fraction: A Randomized Exercise Trial. Arch Phys Med Rehabil. 2017 Mar;98(3):600-603.
3. Meyer K, Schwaibold M, Westbrook S, Beneke R, Hajric R, Lehmann M, Roskamm H. Effects of exercise training and activity restriction on 6-minute walking test performance in patients with chronic heart failure. Am Heart J. 1997 Apr;133(4):447-53.
4. Qu J, Shi H, Guo Y, Chen X, Xiao X, Zheng X, Cui Y. Is the six-minute walk test still reliable compared to cardiopulmonary exercise test for exercise capacity in children with congenital heart disease? Front Pediatr. 2022 Nov 14;10:965739.
5. Ritt LEF, Darzé ES, Feitosa GF, Porto JS, Bastos G, Albuquerque RBL, Feitosa CM, Claro TC, Prado EF, Oliveira QB, Stein R. The Six-Minute Step Test as a Predictor of Functional Capacity according to Peak VO2 in Cardiac Patients. Arq Bras Cardiol. 2021 May;116(5):889-895.
6. Węgrzynowska-Teodorczyk K, Mozdzanowska D, Josiak K, Siennicka A, Nowakowska K, Banasiak W, Jankowska EA, Ponikowski P, Woźniewski M. Could the two-minute step test be an alternative to the six-minute walk test for patients with systolic heart failure? Eur J Prev Cardiol. 2016 Aug;23(12):1307-13.

**Table S2.** CPET and 6MWT protocols in each study.

| **Reference** | **Gold standard** | **Index test** |
| --- | --- | --- |
| Cavero-Redondo et al. (22) | Described in methodology | Described in methodology |
| Costa et al. (32) | CPET was performed on a treadmill using the MetaLyzer 3B gas analyser (Cortex Medical, Leipzig, Germany) | 6MWT standard. Two tests were performed 15 minutes apart and the best test was considered |
| Kehmeier et al. (33) | The CPET was performed on a bicycle in the semi-recumbent training position, within 3 weeks but more than 4 hours after the 6MWT. They started with a 3-minute period at 0 watts, followed by a 25-watt workload increased by 25 watts every two minutes. Participants were monitored with a 12-lead ECG recorded every minute. Blood pressure was taken every 2 minutes. Blood gas analysis (capillary gas analysis) was performed at maximal exercise when the participant said they needed to stop | 6MWT standard in a 30 metre corridor. Participants had to walk as fast as possible, although rest periods were allowed |
| Morales et al. (34) | CPET was performed on a bicycle. An unloaded period of 1 minute followed by a 10 W/min period. The Wasserman equations were used for age- and sex-adjusted VO2max. The results were repeated using the second measurement | Lipkin's method. In a corridor of 20 metres. Participants could stop or slow down, but always in the belief that this was the maximum they could do. The test was carried out twice |
| Pulz et al. (35) | Motorised treadmill with breath-by-breath VO2 measurement (Vmax 29c, SensorMedics, US). Data were collected every 20 seconds. A 12-lead ECG was used. Estimated VO2 at anaerobic threshold was obtained using the modified V-slope and ventilation methods. The Borg scale was passed at the end of the test. | 6MWT standard. Participants were monitored with a Holter (Dynamis 3000, Brazil) and a Polar (Polar Accurex Plus, Finland). |

**Table S3.** Results of the studies included in the systematic review.

| **Reference** | **Cut-off 6MWT** | **Sensitivity** | **Specificity** | **AUC** | **PLR** | **NLR** |
| --- | --- | --- | --- | --- | --- | --- |
| Cavero-Redondo et al. (22) | 359 | 0.75 (0.39, 1.00) | 0.80 (0.43, 1.00) | 0.76 (0.54, 0.99) | 3.75 (0.33, 42.47) | 0.31 (0.03, 3.45) |
| Costa et al. (32) | 520 | 0.75 (0.57, 0.93) | 0.71 (0.53, 0.89) | 0.77 (0.59, 0.95) | 2.59 (0.21, 31.42) | 0.35 (0.03, 4.39) |
| Kehmeier et al. (33) | 482 | 0.79 (0.68, 0.90) | 0.76 (0.65, 0.87) | 0.87 (0.76, 0.98) | 3.29 (0.30, 36.47) | 0.28 (0.02, 3.11) |
| Morales et al. (34) | 450 | 0.80 (0.64, 0.96) | 0.83 (0.67, 0.99) | 0.83 (0.67, 0.99) | 4.71 (0.46, 48.64) | 0.24 (0.02, 2.46) |
| Pulz et al. (35) | 490 | 0.83 (0.69, 0.97) | 0.83 (0.69, 0.97) | 0.89 (0.75, 1.00) | 4.88 (0.49, 48.61) | 0.20 (0.02, 2.04) |

**Figure S1.** Linear regression between CPET and 6MWT scores.


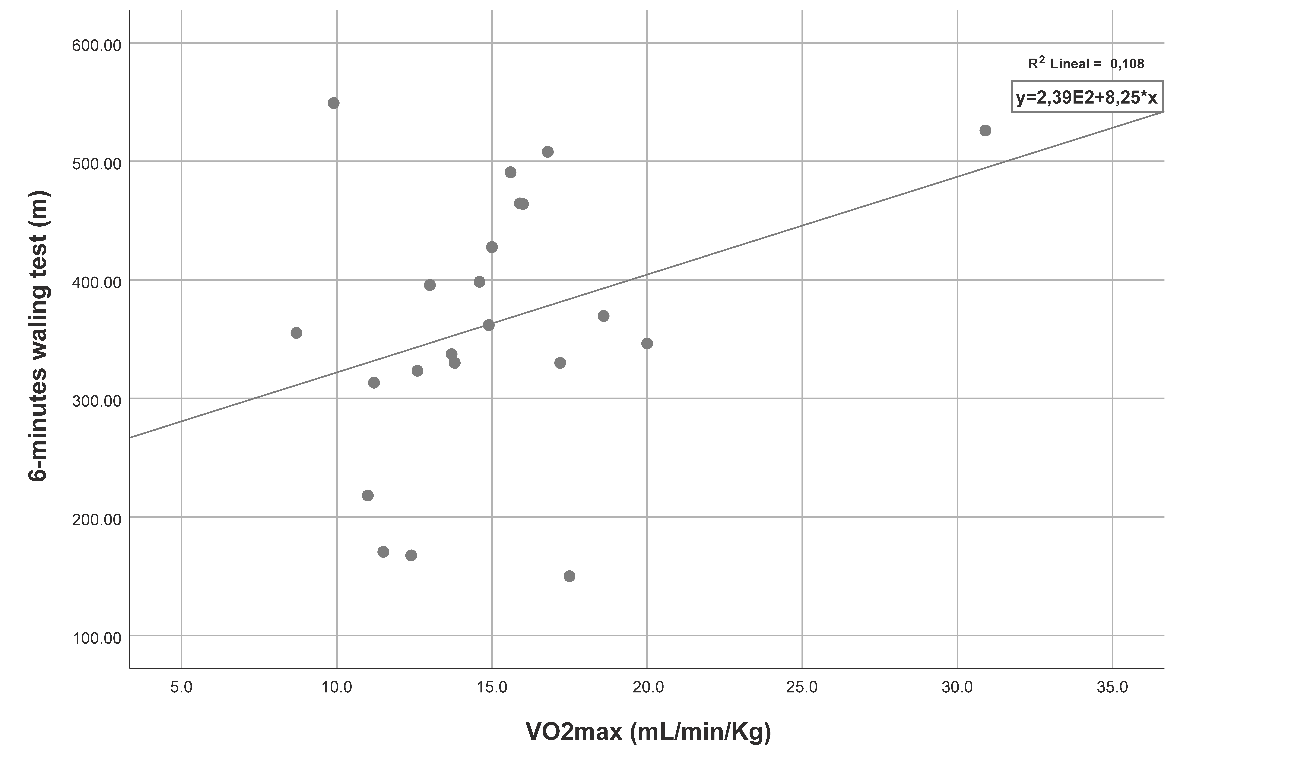


**Figure S2.** PRISMA flowchart of study selection.


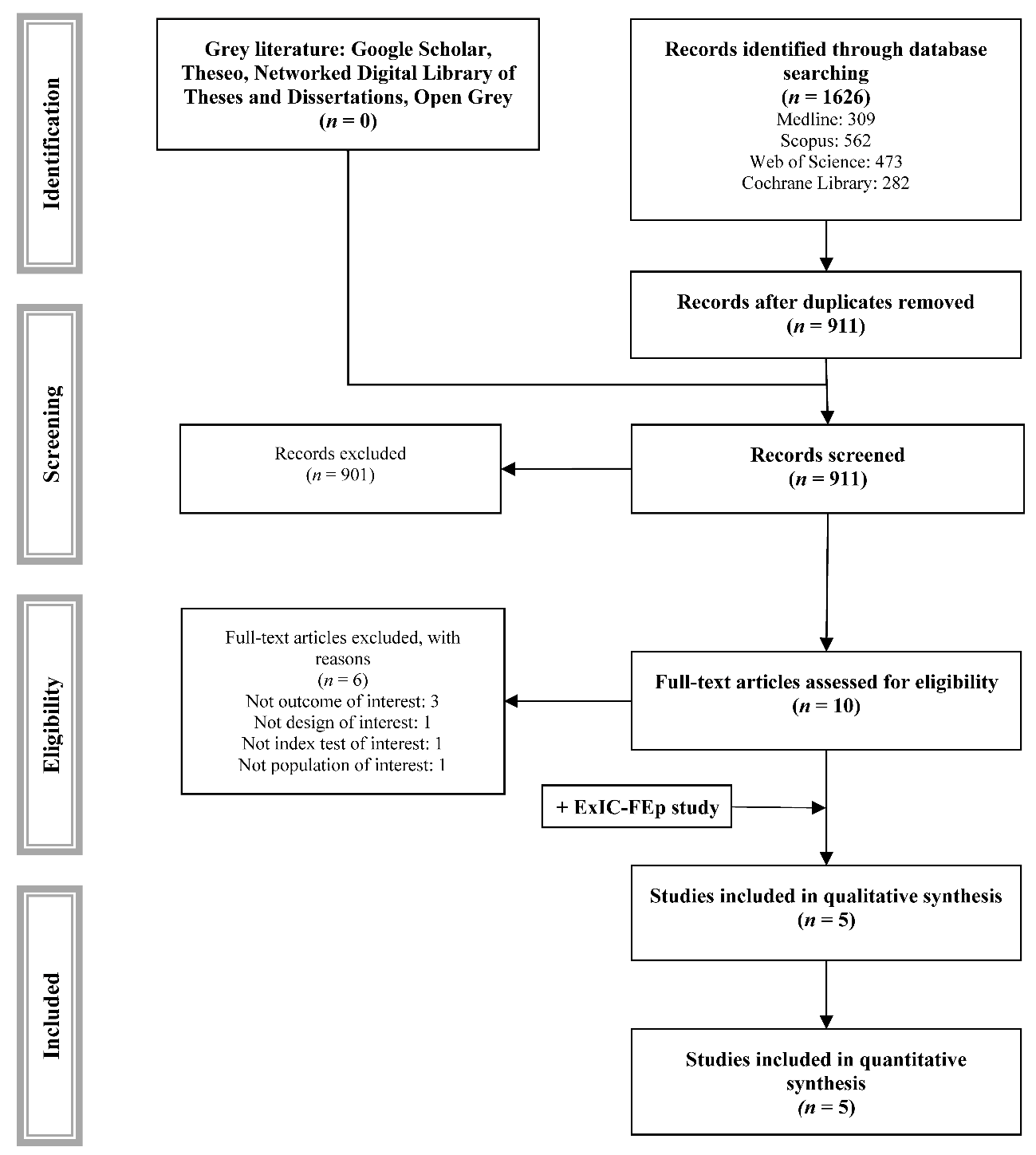


**Figure S3.** Risk of bias assessment.


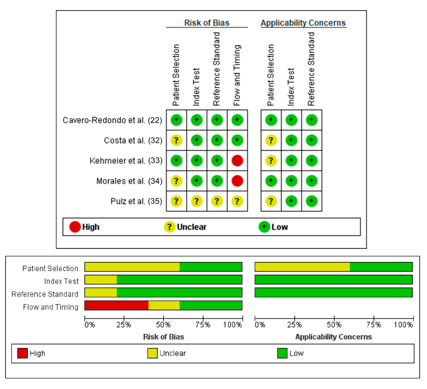


**Figure S4.** Publication bias assessment of sensitivity.


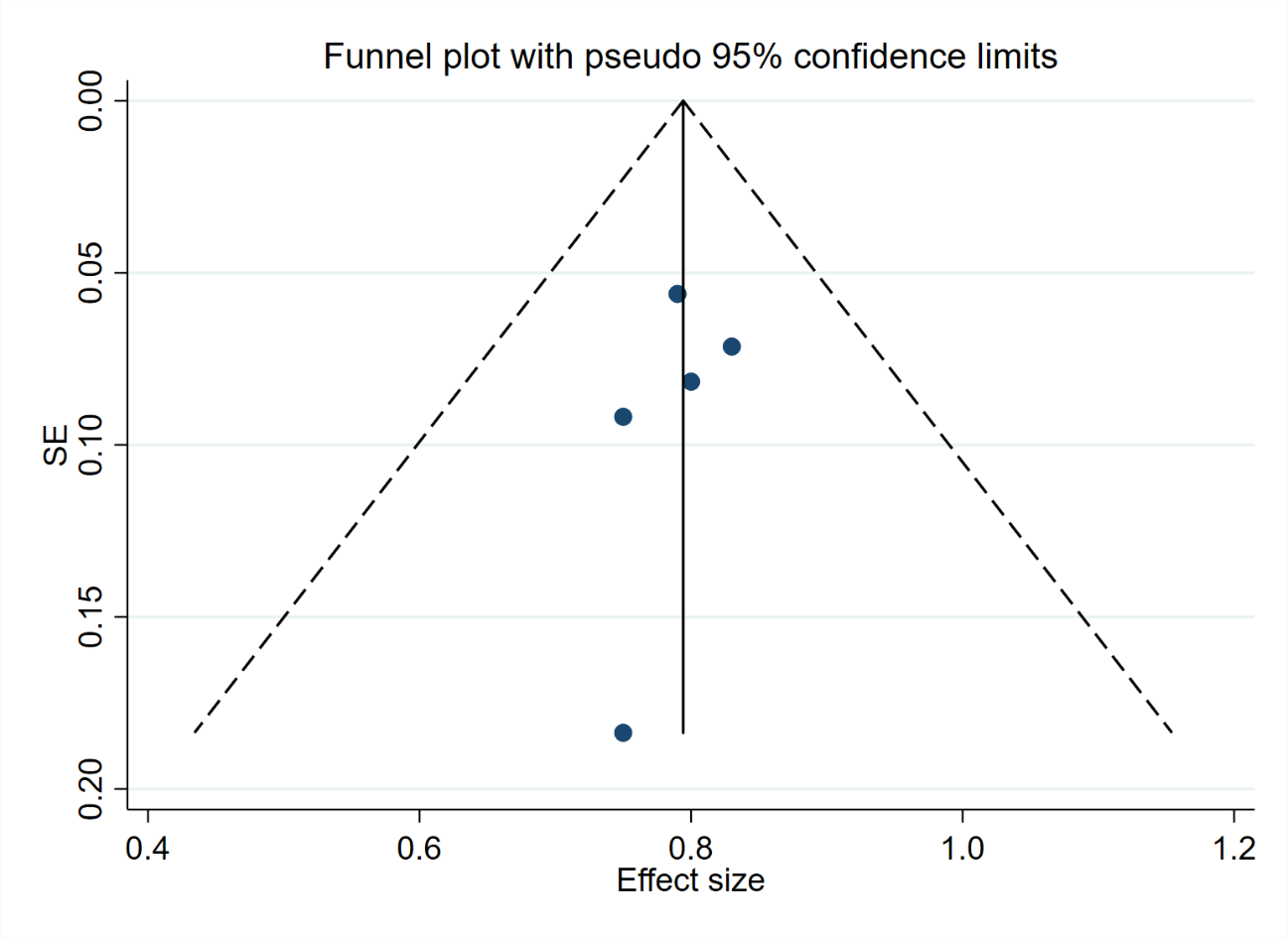


**Figure S5.** Publication bias assessment of specificity.


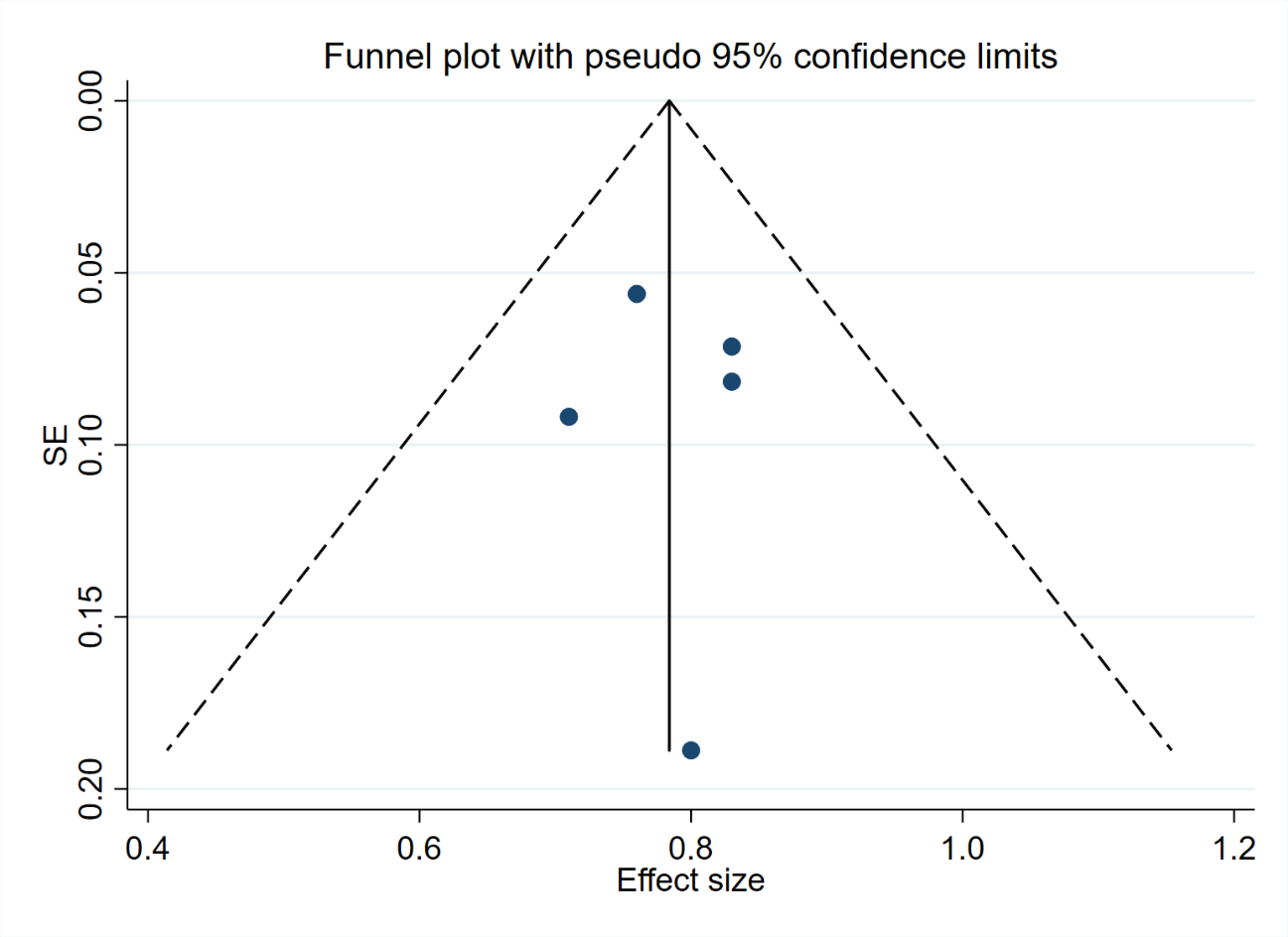


**Figure S6.** Publication bias assessment of area under curve (AUC).


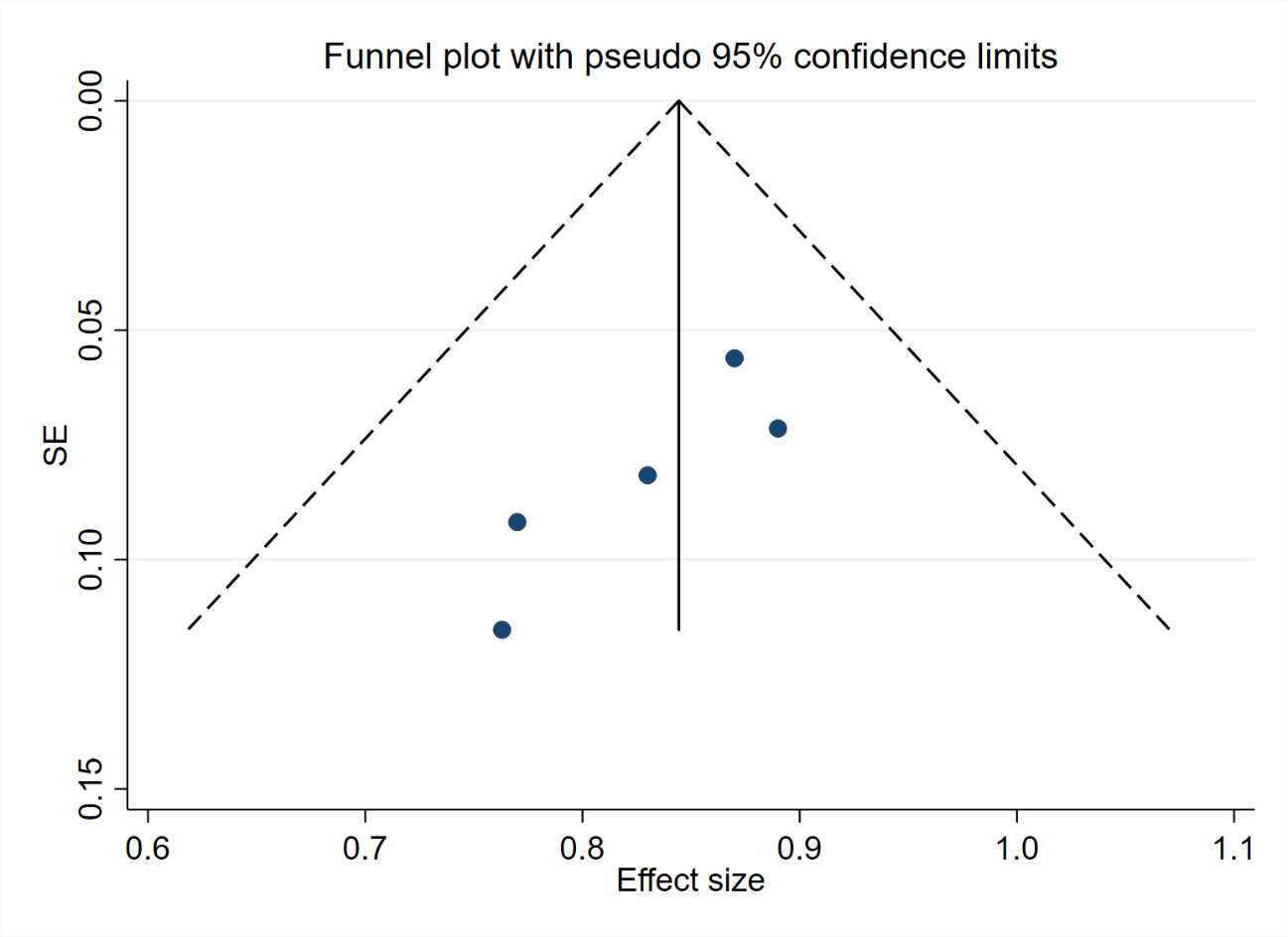


**Figure S7.** Publication bias assessment of positive likelihood ratio (PLR).


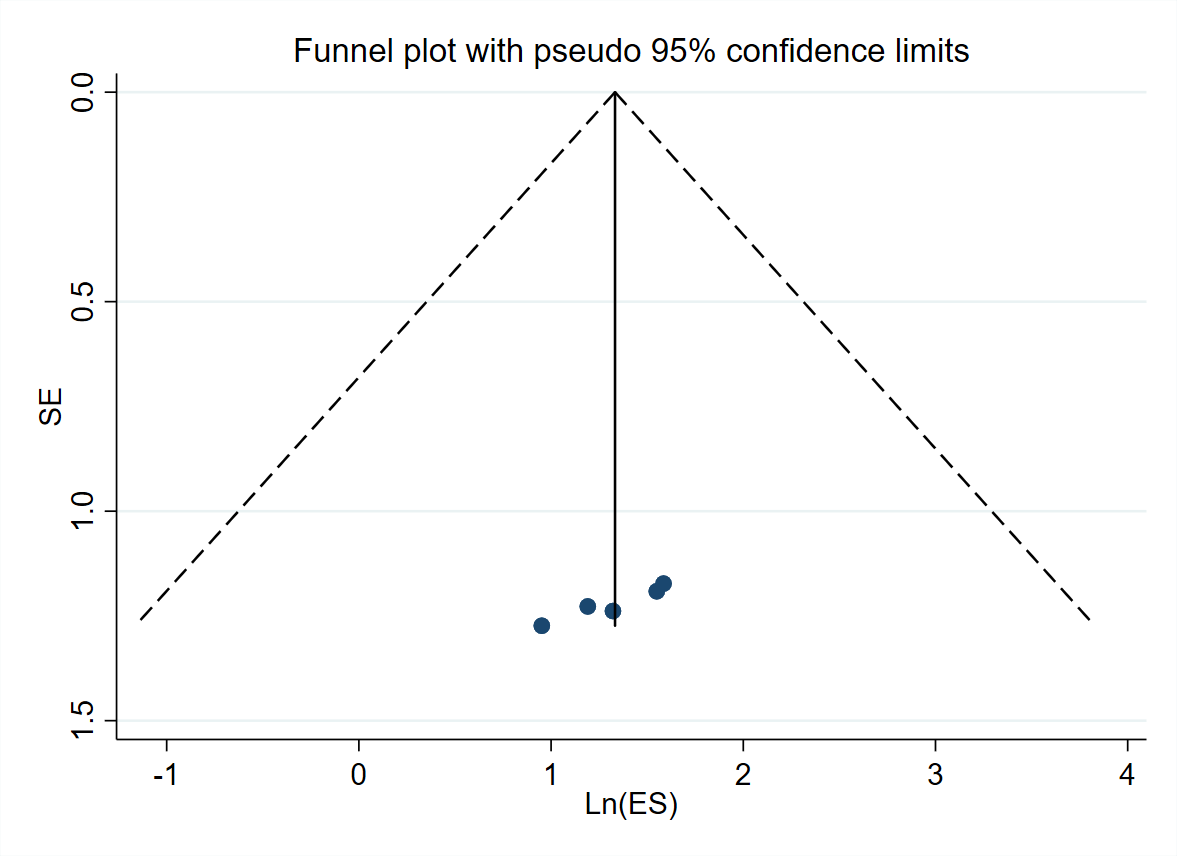


**Figure S8.** Publication bias assessment of negative likelihood ratio (NLR).


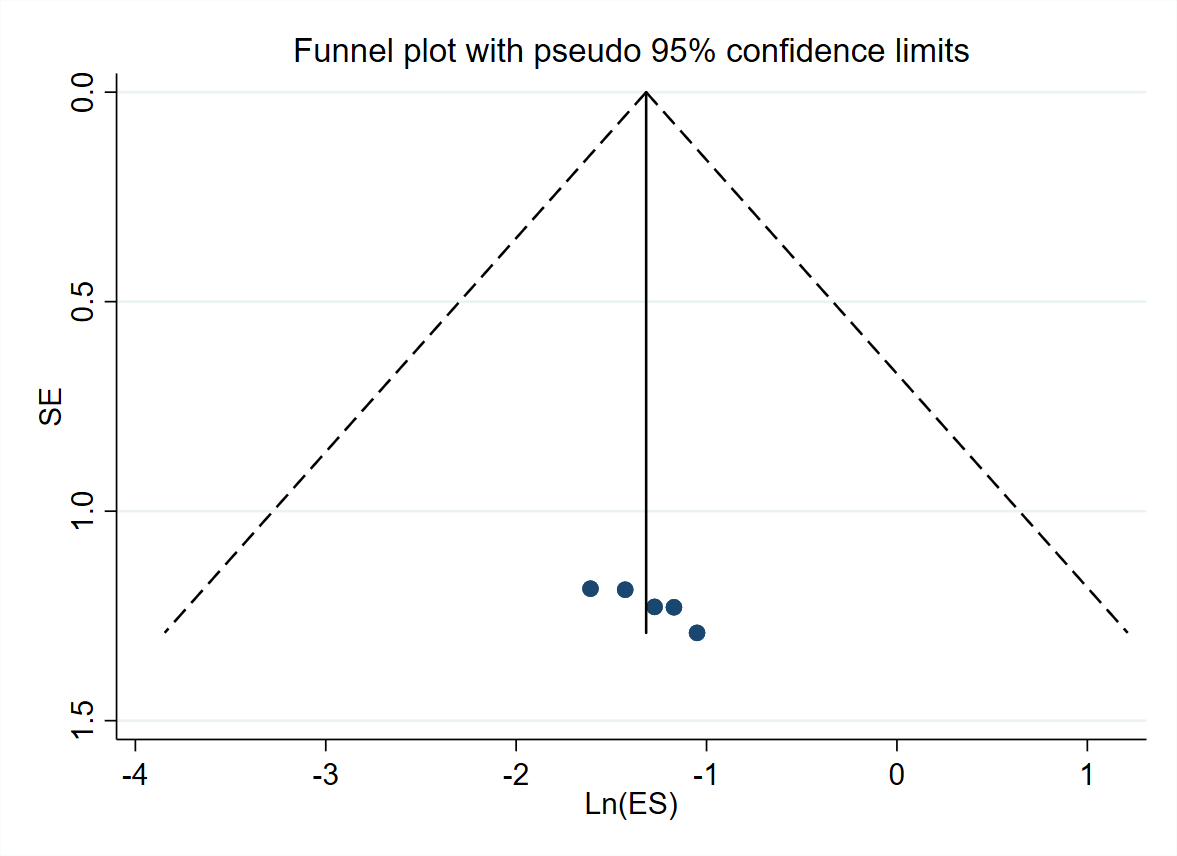


**Appendix S1.** Search strategy.

- Medline, Scopus, Web of Science, Cochrane Library

("heart failure" OR "heart disease" OR "coronary artery disease" OR "cardiovascular disease*") AND ("vo2peak" OR "peak oxygen" OR "peak VO2" OR "peak oxygen uptake" OR "peak oxygen consumption") AND ("six-minute walking test" OR "walk* test*" OR "six-minute" OR "6MWD" OR "6MWT")

- Grey literature

Not specified
